# Identification of Additional Anti-Persister Activity against *Borrelia burgdorferi* from an FDA Drug Library

**DOI:** 10.3390/antibiotics4030397

**Published:** 2015-09-16

**Authors:** Jie Feng, Megan Weitner, Wanliang Shi, Shuo Zhang, David Sullivan, Ying Zhang

**Affiliations:** Department of Molecular Microbiology and Immunology, Bloomberg School of Public Health, Johns Hopkins University, Baltimore, MD 21205, USA; E-Mails: jfeng16@jhu.edu (J.F.); mweitne1@jhu.edu (M.W.); wshi3@jhu.edu (W.S.); shuozhang66@gmail.com (S.Z.); dsulliv7@jhmi.edu (D.S.)

**Keywords:** *Borrelia burgdorferi*, persisters, anti-persister activity, FDA drug library

## Abstract

Lyme disease is a leading vector-borne disease in the United States. Although the majority of Lyme patients can be cured with standard 2–4 week antibiotic treatment, 10%–20% of patients continue to suffer from prolonged post-treatment Lyme disease syndrome (PTLDS). While the cause for this is unclear, persisting organisms not killed by current Lyme antibiotics may be involved. In our previous study, we screened an FDA drug library and reported 27 top hits that showed high activity against *Borrelia* persisters. In this study, we present the results of an additional 113 active hits that have higher activity against the stationary phase *B. burgdorferi* than the currently used Lyme antibiotics. Many antimicrobial agents (antibiotics, antivirals, antifungals, anthelmintics or antiparasitics) used for treating other infections were found to have better activity than the current Lyme antibiotics. These include antibacterials such as rifamycins (3-formal-rifamycin, rifaximin, rifamycin SV), thiostrepton, quinolone drugs (sarafloxacin, clinafloxacin, tosufloxacin), and cell wall inhibitors carbenicillin, tazobactam, aztreonam; antifungal agents such as fluconazole, mepartricin, bifonazole, climbazole, oxiconazole, nystatin; antiviral agents zanamivir, nevirapine, tilorone; antimalarial agents artemisinin, methylene blue, and quidaldine blue; antihelmintic and antiparasitic agents toltrazuril, tartar emetic, potassium antimonyl tartrate trihydrate, oxantel, closantel, hycanthone, pyrimethamine, and tetramisole. Interestingly, drugs used for treating other non-infectious conditions including verteporfin, oltipraz, pyroglutamic acid, pidolic acid, and dextrorphan tartrate, that act on the glutathione/γ-glutamyl pathway involved in protection against free radical damage, and also the antidepressant drug indatraline, were found to have high activity against stationary phase *B. burgdorferi.* Among the active hits, agents that affect cell membranes, energy production, and reactive oxygen species production are more active against the *B. burgdorferi* persisters than the commonly used antibiotics that inhibit macromolecule biosynthesis. Future studies are needed to evaluate and optimize the promising active hits in drug combination studies *in vitro* and also *in vivo* in animal models. These studies may have implications for developing more effective treatments of Lyme disease.

## 1. Introduction

*Borrelia burgdorferi* is the causative agent of Lyme disease, the most common vector-borne disease in the United States and Europe. Although about 27,000 confirmed cases of Lyme disease in the United States were reported to the Centers for Disease Control and Prevention (CDC) in 2013, the total number of cases is estimated to be as high as 300,000 each year [1,2]. *B.* *burgdorferi* is transmitted during the blood feeding of Ixodes ticks on hosts including rodents, small mammals, and humans [3]. Lyme disease in humans is a multi-system disorder whose early stage is characterized by erythema migrans, a rapidly spreading rash that appears at the cutaneous site of infection in about 50% of patients [4]. Upon bacterial dissemination, patients can experience severe symptoms such as arthritis, carditis, and neurologic impairment [4].

The current treatment for Lyme disease is a 2–4 week antibiotic monotherapy with doxycycline, amoxicillin, or cefuroxime axetil [4]. However, according to the CDC, about 10%–20% of patients receiving this treatment experience chronic symptoms such as fatigue, muscle pain, and neurological impairment even six months after treatment [5], but a more recent study estimated the percentage of such patients to be at least 20% [6]. Patients with these symptoms are diagnosed with Post-Treatment Lyme Disease Syndrome (PTLDS) and report significantly impaired functional ability and lower quality of life compared to Lyme patients without these symptoms [7]. The cause of PTLDS is unknown. Several theories have been proposed to explain this syndrome, including host response to continued presence of bacterial debris, autoimmunity, co-infections, and bacterial persisters not killed by the current Lyme antibiotics [8].

Evidence that supports the continued presence of persisting organisms despite antibiotic treatment has been well documented in various animal models such as mice, dogs, and nonhuman primates [9,10,11,12]. Intriguingly, the organism could not be cultured in conventional culture medium after antibiotic treatment but could be detected by more sensitive and indirect techniques such as xenodiagnosis and PCR. Similarly, in patients with chronic Lyme infections, signs of persisting organisms in a nonculturable form could be detected by positive PCR and xenodiagnosis [13]. Persistent bacteria are suggested as an explanation for the chronic symptoms of PTLDS as well as the observations of *B. burgdorferi* DNA without positive culturing results [14,15].

Persisters are a minor population of non-growing bacterial cells that are not killed by bactericidal antibiotic treatment [16,17]. Persisters are a heterogeneous bacterial population that are genetically drug susceptible but have phenotypic variations which allow them to survive in the presence of stressors such as antibiotics [17]. *B. burgdorferi* can change morphologies and develop persisters as the culture ages [18,19]. The log phase culture of *B. burgdorferi* consists primarily of spirochetes but round bodies and microcolonies become more abundant as the culture reaches stationary phase [18,19]. The current Lyme antibiotics, while having high activity against the spirochete log phase bacteria, show little activity against the stationary phase morphological variants which show features of persisters [18,19,20].

To identify drugs that target *B. burgdorferi* persisters, we recently screened a Food and Drug Administration (FDA) drug library and identified 165 hits with higher activity against *B. burgdorferi* persisters than the currently used Lyme antibiotics amoxicillin and doxycycline [18]. In that study, we reported the top 27 hits and some top hits were further evaluated in drug combination studies [18,19]. In this study, we present findings on the remainder of the 113 drug candidates from the FDA drug library with higher activity against the *B. burgdorferi* stationary phase culture than amoxicillin and doxycycline.

## 2. Results and Discussion

### 2.1. Identification of Drug Candidates with High Anti-Persister Activity

In our previous study, we identified 165 hits with higher activity against *B. burgdorferi* persisters than the currently used Lyme antibiotics. In this study, we further analyzed and characterized these active hits. By removing redundant hits from the library and also those that could not be repeated, we obtained 113 that gave consistent results (Supplementary Table S1). Of the 113 hits, the top 52 candidates that can be used in humans and killed 65% or more of the stationary phase bacteria according to either the SYBR Green I/Propidium Iodide (PI) assay or microscopic quantitation are presented in Table 1. The remainder of active hits that may not be used in humans and also less active ones are presented in Supplementary Table S1. However, these agents in general were not as active as the top hits such as daptomycin, clofazimine, cefoperazone, and anthracyclines from the previous screens [18,21]. These active hits are grouped into antimicrobial agents (antibiotics, antivirals, antifungals, anthelmintics or antiparasitics), agents used for treating other disease conditions, as well as agents that may only be used as topical agents or not used internally and are presented below (Table 1).

We used a relatively high drug concentration of 50 μM in our drug screens with seven-day-old stationary phase cultures; as in our preliminary pilot screens, lower drug concentrations, such as 10 μM, commonly used for actively growing organisms or protein target screens, would not allow one to see the effect of any compounds—even the highly active compounds such as daptomycin—under this condition. The findings with 50 μM are still valid and relevant, because in the setting of drug combinations, the persister-active hits can be used at lower clinically relevant concentrations (10 μg/mL for daptomycin) and still allow one to see the effect and importance of the persister-active hits in eradicating more resistant microcolony forms of *B. burgdorferi* persisters [19]. In addition, taking into consideration some drugs such as pyrazinamide, with high activity against persisters *in vivo* but poor activity *in vitro* [17], we used this higher concentration to increase the sensitivity of the screen. We plan to validate these results using clinically relevant dosages in future drug combination studies *in vitro* and *in vivo*.

**Table 1:** Top 52 active hits with better activity (*p*-value < 0.05 or live percentage by microscopy assay less than 68%) against *B. burgdorferi* stationary phase cells than current Lyme antibiotics ^a^.

Drugs (50 μM)	Category	Residual Viable Cells (Microscopy) ^b^	Residual Viable Cells (SYBR Green/PI) ^c^	*p*-Value ^d^
Control (no drug)		93%	94%	-
Doxycycline	Lyme antibiotic	75%	67%	0.23360
Amoxicillin	Lyme antibiotic	76%	76%	1.00000
Cefuroxime	Lyme antibiotic	49%	43%	0.00032
Daptomycin	Antibiotic	35%	28%	0.00001
*Verteporfin* ^e^	Ophthalmic	*47%*	*27%*	*0.00284*
3-formyl Rifamycin	Antibacterial	59%	42%	0.00103
Tartar emetic	Anthelmintic	45%	42%	0.00250
Toltrazuril	Antiprotozoal	60%	43%	0.00296
Thiostrepton	Antibiotic	66%	43%	0.00131
Mepartricin	Antifungal	60%	43%	0.03214
Tilorone	Antiviral		44%	0.04955
Oxantel	Anthelmintic	63%	44%	0.01599
*Pidolic acid* ^e^	*Antiseptic*	*45%*	*45%*	*0.00477*
Hycanthone	Anthelmintic		45%	0.00154
Pyrimethamine	Antiprotozoal	55%	45%	0.00030
Carbenicillin	Antibiotic	64%	46%	0.06453
*Oltipraz*	*Antitumor*	*55%*	*46%*	*0.00121*
Bitoscanate	Anthelmintic		46%	0.00730
Sarafloxacin	Antibiotic	50%	47%	0.05063
Bacitracin	Antibiotic	60%	47%	0.06536
*Dextrorphan tartrate* ^e^	*Analgesic*	*43%*	*47%*	0.00361
Tetramisole	Anthelmintic		48%	0.07051
Bifonazole	Antifungal	50%	48%	0.09243
Ethacridine lactate	Antiseptic		48%	0.04619
Zanamivir	Antiviral	60%	49%	0.01224
Artemesinin	Antimalarial	45%	49%	0.10432
Oxibendazole	Anthelminthic		51%	0.00895
*Indatraline* ^e^	*Antidepressant*	*43%*	*51%*	0.02578
Nevirapine	Antiviral		51%	0.01604
Ganciclovir	Antiviral		53%	0.04466
Phenothiazine	Anthelminthic	53%	54%	0.00228
Oxfendazole	Anthelminthic		54%	0.00095
Flubendazole	Anthelminthic		54%	0.01183
Tazobactam	Antibiotic	56%	54%	0.10052
Aztreonam	Antibiotic	50%	55%	0.08105
Benzoylpas	Antibiotic		55%	0.04017
Fluconazole	Antifungal	45%	55%	0.10643
Cefixime	Antibiotic	56%	56%	0.03238
Sulfamoxole	Antibiotic	55%	57%	0.02541
Tosufloxacin	Antibiotic		57%	0.00067
Lamivudine	Antiviral		58%	0.01121
Cefsulodin	Antibiotic		60%	0.01463
Didanosine	Antiviral		61%	0.02494
Floxuridine	Antiviral		61%	0.01935
Cyacetacide	Antibacterial		61%	0.03407
Oxiconazole nitrate	Antifungal		62%	0.04957
Roxithromycin	Antibiotic	65%	62%	0.15382
Ribavirin	Antiviral		63%	0.00801
Griseofulvin	Antifungal		63%	0.00957
Rifamycin sv	Antibiotic	60%	63%	0.01872
Penciclovir	Antiviral	60%	64%	0.09707
Nystatin	Antifungal		64%	0.03312
Penimepicycline	Antibiotic	60%	65%	0.00972
Puromycin	Antibiotic	48%	65%	0.29297
Quinaldine blue	Antimalarial	35%	Over range ^f^	-
Methylene blue hydrate	Antimethemoglobinemic	40%	Over range ^f^	-

^a^ Stationary phase *B. burgdorferi* (seven day old) cells were treated with drugs for seven days. Daptomycin was used as a positive control with known high activity against *B. burgdorferi* persisters as shown previously. Drugs with live percentage of *B. burgdorferi* less than 65% by microscopy after drug exposure are presented in the table; ^b^ Residual viable *B. burgdorferi* was assayed by epifluorescence microscope counting; ^c^ Residual viable *B. burgdorferi* was calculated according to the regression equation and ratio of green/red fluorescence obtained by SYBR Green I/PI assay. Three images of each sample were captured and quantitatively analyzed to determine the mean percent residual cells as indicated; ^d^ *p*-values of the standard t-test for the treated groups (*n* = 3) *vs.* a control group treated with amoxicillin, which is known to have poor activity against stationary-phase persisters; ^e^ The italicized drugs are used to treat disease other than infection; ^f^ The value is over that of the drug-free control due to color of the compounds.

In our previous study, we compared the activity of antibiotics against non-growing persisters with their activity against growing *B. burgdorferi* [18]. Here we also tested the minimum inhibitory concentration (MIC) of some active hits. We found these drugs showed good activity against the growing *B. burgdorferi* with low MICs (Table 2). The maximum blood drug concentrations (Cmax) of several active hits, including 3-formyl rifamycin, oltipraz, and fluconazole, were higher than the MICs of these drug candidates, indicating these drugs are likely to achieve clinically relevant drug concentrations. However, some drugs such as verteporfin, pidolic acid, and dextrorphan tartrate had lower Cmax values than their corresponding MICs. Some other active hits such as thonzonium bromide, benzododecinium chloride, and quinaldine blue did not have Cmax values as they are used only topically or not available.

**Table 2:** Minimum inhibitory concentration values of some persister-active hits for *B. burgdorferi.*

Active Hits	MIC (μg/mL)	Cmax (μg/mL)
Verteporfin	4.49–8.98	1.03–1.14
Thonzonium Bromide	0.92–1.85	NA
Benzododecinium Chloride	0.60–1.20	NA
3-formyl Rifamycin	2.27–4.54	10
Pidolic Acid	0.81–1.61	0.024
Oltipraz	0.71–1.41	4.97
Fluconazole	0.48–0.96	1.48–11.9
Dextrorphan Tartrate	1.27–2.55	0.025–0.030
Quinaldine Blue	2.43–4.86	NA

NA: not available.

### 2.2. Antimicrobial Agents with High Activity against Stationary Phase B. burgdorferi

**Figure 1:**
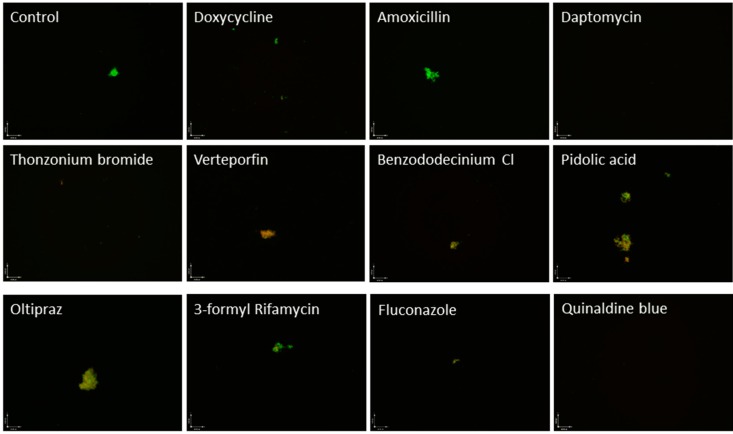
Image of *B. burgdorferi* stationary phase culture (seven day old) incubated for seven days with the indicated drugs, stained by SYBR Green I/PI assay, and examined using epifluorescence microscopy. Live cells are indicated by green fluorescence and dead cells are indicated by red fluorescence.

Readily available drugs with low toxicity are important objectives in this screen as they are the most likely to be used for the clinical treatment of Lyme disease. Here we focused on antimicrobial agents used in humans that had higher activity against the stationary phase *B. burgdorferi* than the commonly used Lyme antibiotics. The antibacterial agents include rifamycins (3-formal-rifamycin, rifaximin, rifamycin SV) (Figure 1), thiostrepton, quinolone drugs (sarafloxacin, clinafloxacin, tosufloxacin), carbenicillin, tazobactam, aztreonam, and puromycin (Table 1, Supplementary Table S1). Some antifungal agents such as fluconazole (Figure 1), mepartricin, bifonazole, climbazole, oxiconazole, and nystatin had reasonable activity against stationary phase *B. burgdorferi* (Table 1, Supplementary Table S1). Antiviral agents zanamivir, nevirapine, and tilorone (orally active interferon inducer) had good activity against stationary phase *B. burgdorferi.* Antimalarial agents artemisinin, methylene blue, and quidaldine blue were found to have good activity against stationary phase *B. burgdorferi* (Table 1). Antihelmintic and antiparasitic agents that had activity against *B. burgdorferi* included toltrazuril, tartar emetic, potassium antimonyl tartrate trihydrate, oxantel, closantel, hycanthone, pyrimethamine, and tetramisole (Table 1). These drugs with high activity against stationary phase *B. burgdorferi* *in vitro* are good potential candidates for drug combination studies and for further evaluation in animal models.

As previously published, the SYBR Green I/PI assay is a high-throughput technique that uses the ratio of green:red fluorescence in each sample to quantitate the amount of residual viable cells remaining [18]. While this technique has the benefits of high-throughput analysis, discoloration of the culture medium by test drugs can result in altered readings [18]. Quinaldine blue and methylene blue are two drugs whose staining properties resulted in medium discoloration and required verification through microscopy. Careful microscopy analysis revealed that quinaldine blue and methylene blue had high activity against *B. burgdorferi* persisters (Table 1, Figure 1). Quinaldine (2-methylquinoline) is a heterocyclic quinoline compound that is used as an antimalarial and in dye manufacturing, food colorants, pH indicators, and pharmaceuticals. Methylene blue was originally used as an antimalarial and is used to treat methemoglobinemia and urinary tract infections.

Zanamivir is a clinically used antiviral agent that inhibits the neurominidase inhibitor that is inhaled as an aerosol to shorten the duration of influenza infections by preventing neuraminidase from releasing virions from the infected cells [22]. Recently, multiple bacterial species have been shown to express bacterial neuraminidases capable of cleaving α2,3-sialic acids [23]. These neuraminidases have been implicated in biofilm formation, with a *P. aeruginosa* neuraminidase mutant showing decreased ability to colonize the mouse respiratory tract and decreased biofilm production [23]. It remains to be seen if zanamivir acts in a similar manner in *B. burgdorferi*.

### 2.3. Agents Used for Treating Other Disease Conditions

It is interesting to note that several highly active drugs identified in our screen, including verteporfin, oltipraz, pyroglutamic acid, pidolic acid (Figure 1), and dextrorphan tartrate, act on the glutathione/γ-glutamyl pathway used in mammalian cells which involved in protection against intracellular damage from free radicals and peroxides. Glutathione (GSH) is a reducing agent produced in the cytoplasm and transferred to the mitochondria by glutathione-S-transferase (GST), where it protects the mitochondria from reactive oxygen species (ROS) damage and functions in amino acid transport [24,25]. Reduced levels of GSH have been linked to increased sensitivity to ROS damage, resulting in mitochondrial swelling and subsequent damage [24,26].

Verteporfin (Visudyne), a benzophorphyrin derivative, is a photosensitizing agent currently used to treat the macular degeneration that affects the γ-glutamyl pathway [24,27,28]. This intravenous drug is transported in oxygenated blood by lipoproteins, and is activated by laser light treatment allowing for precise chemotherapeutic application [24]. Verteporfin is a possible effector of cell membrane permeability through ROS lipid peroxidation [24,29]. Activated verteporfin has been shown to target the mitochondria, producing reactive oxygen radicals and nitric oxide that damage local endothelium and seal leaky vessels [24,27]. Verteporfin depletes GSH levels in HepG2 cells after activation, possibly through increased nitric oxide production [24]. Oltipraz is an organosulfur compound that belongs to the dithiolethione class. It has been shown to inhibit schistosome and prevent the formation of cancer. It activates phase II detoxification enzymes in mammalian cells, which results in the binding of glutathione to electrophilic compounds and subsequent protection against reactive oxygen species (ROS) damage [30,31]. Pyroglutamic acid (PCA) or pidolic acid (pidolate) or 5-oxoproline is an amino acid derivative that is involved in the γ-glutamyl cycle. PCA is a metabolite of the glutathione cycle which is broken down to glutamate and cysteine, which are then converted back into glutathione [26], and is used in humans as dietary supplement and skin moisturizer retainer.

The significant number of highly active drugs that act upon this γ-glutamyl pathway suggests that this pathway is important for persisters and that ROS and peroxide-induced damage are important for killing persisters. We anticipate that inhibition of this pathway could be a good therapeutic target for *B. burgdorferi* persisters for the improved treatment of Lyme disease. Future studies are needed to further evaluate if these agents are active in drug combination studies and in animal models.

### 2.4. Active Hits that Are Topical Agents or Toxic for Internal Use

Thonzonium bromide, benzododecinium chloride, and butyl chloride were found to have very high activities against stationary phase *B. burgdorferi* (Supplementary Table S1, Figure 1). Thonzonium bromide even had comparable activity to daptomycin against stationary phase *B. burgdorferi*. However, thonzonium bromide is a cationic detergent and surfactant that is used as a topical agent in combination with other compounds to assist in the penetration of cellular membranes [32]. Thonzonium bromide has been shown to inhibit vacuolar ATPases in yeast, which is an enzyme that is closely related to the ATPase found in *B. burgdorferi* [33,34,35]. In *C. albicans,* thonzonium bromide was also shown to inhibit ATPases in isolated vacuoles and cause general cellular toxicity [33,34]. Thonzonium bromide was also shown to be active against preformed *C. albicans* biofilms [32]. Benzododecinium chloride is a C12-substituted alkyl chain derivate of the quarternary ammonium detergent benzalkonium chloride that alters cell membrane permeability and can cause cell lysis through lipid dispersion [36,37]. Benzododecinium chloride was shown in *S. aureus* to have higher activity against the biofilm form of the bacteria than the free planktonic form *in vitro* [38]. Since thonzonium bromide and benzododecinium chloride have strong detergent properties causing generalized cellular damage in humans, they may not be used directly for Lyme treatment. However, the high activity of these drugs against *B. burgdorferi* persisters suggests that both the cell membrane and biofilms are potential targets for future persister drug design.

It is worth noting that the most active hits from the compound library screen are those that affect cell membranes (benzododecinium chloride, thonzonium bromide, zanamivir). This is consistent with our previous finding that daptomycin and clofazimine may act on the cell membrane to show their high activity against *B. burgdorferi* persisters [18]. Indeed, agents that target bacterial cell membranes have been found to be active against persisters in different bacterial pathogens such as *M. tuberculosis* and *E. coli* [39,40,41]. Other active hits that show good activity against *B. burgdorferi* persisters interfere with energy production (thonzonium bromide, oxantel) and ROS production (verteporfin, oltipraz, pyroglutamic acid, pidolic acid). Our data showed that these three types of agents, cell membrane disruptors, energy inhibitors, and ROS producers, are generally more active against the *B. burgdorferi* persisters than the more conventional antibiotics that inhibit cell wall, protein, RNA, and DNA syntheses (Table 1, Supplementary Table S1). Future studies are needed to assess the activity of these agents in combination with Lyme antibiotics for more effective eradication of *B. burgdorferi* persisters *in vitro* [19].

Efflux pumps of *B burgdorferi* persisters have not received any attention other than in the structural study of the adaptor protein of the tripartite efflux pump of the organism. Given our results in this study and also our recent observation that efflux and transporters are upregulated in *B. burgdorferi* persisters [42], it is very likely that over-expressed efflux pumps are one of the causes for the persisters and that compounds known to inhibit bacterial efflux pumps such as RND proton motive force-dependent pumps are possible lead compounds for the development of effective drugs for the treatment of persisting Lyme disease.

A recent study by Sharma *et al.* used colony-forming unit (CFU) assay to assess “persisters” in *B. burgdorferi* [43]. However, the CFU assay, while working well for bacteria that easily form colonies on agar plates, does not work well for *B. burgdorferi* and is not commonly used for viability assessment for *B. burgdorferi.* Instead, BacLight LIVE/DEAD viability assay is the most commonly used method for assessing drug activity in this field. In addition, it is well known that true *B. burgdorferi* persisters after antibiotic treatment *in vivo* cannot be cultured in Barbour-Stoenner-Kelly (BSK) media, and therefore the CFU assay may not be an appropriate method to evaluate the survival of persisters from *in vivo*. Furthermore, the CFU assay may be used only on relatively young cultures (3–5 days) that consist primarily of planktonic spirochetal forms, but cannot be used for older true stationary phase cultures (7–10 days) that consist mainly of aggregated microcolonies [19]. Therefore, we did not use CFU assay in our studies involving seven-day-old cultures, but instead used SYBYR Green I/PI viability assay, which is similar in principle but more sensitive than BacLight LIVE/DEAD viability assay [44]; we also used subculture in liquid medium to assess the anti-persister activity of the drugs. In contrast to the CFU assay, the SYBYR Green I/PI viability assay and subculture in liquid medium can be readily used and are more suited to this setting. In addition, the CFU assay cannot be used for drug screens as it is not only infeasible due to the washing to remove drugs for the CFU assay in a high-throughput format, but it also requires a significant amount of drugs, which precluded us from testing the active hits with the CFU assay, as the FDA drug library did not have a sufficient amount for it. Nevertheless, the CFU assay will be performed in future studies on selected active hits using younger cultures with primarily planktonic spirochetes when a sufficient quantity of the drugs is secured for testing.

Drug screens are subject to false positive non-specific hits referred to as pan-assay interference compounds (PAINS) in protein target-based screens [45]. However, because this study used whole organism (stationary phase culture)-based screens, most of the factors that can lead to PAINS are not present in our assay system. Nevertheless, we looked into the possible presence of PAINS in our active hits derived from the FDA drug library using the software from the Scripps Institute program for chemical promiscuity evaluation [46]. We found the majority of our hits to be highly specific, and the only identified PAINS in our highly active hits included FDA-approved drugs that are also coloring chemicals including methylene blue, quinaldine blue, and ethacridine lactate. The possibility of the PAINS was excluded using manual verification of the anti-persister activity of these drugs through epifluorescence microscopy.

The current study has significant limitations as it looks at the effect of these compounds in a cell-free culture system. Whether these active hits have efficacy *in vivo* is influenced by many factors such as drug absorption, appropriate blood and tissue drug concentrations, tissue penetration and tropism, immune reactivity, and other factors that are missing from the cell-free system. Further studies are needed to confirm if the active hits are effective *in vivo* in animal models before human studies.

## 3. Experimental Section

### 3.1. Strain and Culture Techniques

*Borrelia burgdorferi* strain B31 (ATCC35210) was received from the American Type Tissue Collection (Manassas, VA, USA) and was grown in BSK-H medium (HiMedia Laboratories, Mumbai, India) and 6% rabbit serum (Sigma Aldrich, St. Louis, MO, USA). The culture was filtered and sterilized using a 0.2 mm filter and incubated in capped sterile 50 mL conical tubes (BD Biosciences, San Jose, CA, USA) at 33 °C for seven days without antibiotics until the culture reached stationary phase. Seven-day-old stationary phase cultures were transferred to a 96-well culture plate for evaluation of drugs on active *B. burgdorferi* persisters.

### 3.2. Microscopy

The cultures were examined using a Nikon Eclipse E800 microscope with differential interference contrast and epifluorescent illumination. The pictures were captured using a SPOT slider color camera. A SYBR Green I/PI assay was used to assess the viability of the bacterial sample using the ratio of live to dead *B. burgdorferi* (measured with green and red fluorescence, respectively) as measured by a plate reader. The cellular counts were made by counting 100–200 cells per image based on three images representative of the bacterial samples using epifluorescence microscopy and quantitatively analyzed using Image Pro-Plus software to calculate the fluorescence intensity as described [44].

### 3.3. Drug Library Screens for Activity against B. burgdorferi Persisters in Vitro

The FDA drug library screens against the stationary phase *B. burgdorferi* persister model were performed as described [18]. Briefly, prediluted drug stock (10 µL) was added to seven-day-old stationary phase *B. burgdorferi* culture (90 µL) to achieve a 50 µM final drug concentration per well. The plates were then incubated at 33 °C for seven days, at which point the SYBR/PI rapid viability assay was performed in a fluorescence plate reader to obtain the green-red fluorescence ratio. The top hits from the SYBR Green I/PI assay were then examined using epifluorescence microscopy to ensure accuracy of the SYBR Green I/PI readings and to ensure no fluorescent contamination from colored test drugs as described previously [18].

## 4. Conclusions

In this study we identified 113 active hits that have higher activity against the stationary phase *B. burgdorferi* than the currently used Lyme antibiotics. These active hits include commonly used antimicrobials for treating other infections as well as some agents that are used for treating other disease conditions. Agents that affect cell membranes, energy production, and ROS production are generally more active against the *B. burgdorferi* persisters than the commonly used antibiotics that inhibit macromolecule biosyntheses. Future studies are needed to evaluate and optimize the promising active hits in drug combination studies *in vitro* and also *in vivo* in animal models. These studies may have implications for the improved treatment of Lyme disease.

## Supplementary Files

Supplementary File 1
