# Supplementary material for: Identification of Additional Anti-Persister Activity against Borrelia burgdorferi from an FDA Drug Library
Source: Antibiotics (Basel). 2015 Sep 16;4(3):397–410. doi: 10.3390/antibiotics4030397 (PMC4790293; doi:10.3390/antibiotics4030397)
Supplement: Supplementary File 1 [file antibiotics-04-00397-s001.pdf]

Supplementary Material

Table S1. Active hits that showed better activity against stationary phase *B. burgdorferi* than the current Lyme antibiotics [a].

Drugs (50 µM)	Category	Residual Viable Cells (Microscopy) [b]	Residual Viable Cells (SYBR/PI) [c]
Control		93%	94%
Doxycycline	Lyme antibiotic	75%	67%
Amoxicillin	Lyme antibiotic	76%	76%
Daptomycin	Antibiotic	35%	28%
Verteporfin	Ophthalmic	47%	27%
Thonzonium bromide	Antiseptic	33%	31%
Tetrachloroethylene	Anthelmintic	53%	36%
Benzododecinium chloride	Antiseptic		40%
Butyl chloride (1-Chlorobutane)	Anthelminthic		41%
3-formyl Rifamycin	Antibacterial (tuberculostatic)	59%	42%
Potassium antimonyl tartrate trihydrate	Anthelmintic	45%	42%
Toltrazuril	Antiprotozoal (Coccidostat)		43%
Thiostrepton	Antibiotic	66%	43%
Pyroglutamic acid, DL (DL-2-Pyrrolidone-5-Carboxylic Acid), Pidolic acid	Topical Antiseptic		43%
Mepartricin	Antifungal		43%
Tilorone dihydrochloride	Antiviral		44%
Oxantel	Anthelmintic		44%
Hycanthone	Anthelmintic (Schistosoma)		45%
Pyrimethamine	Antiprotozoal (Toxoplasma)	55%	45%
Trilocarban (3,4,4′-Trichlorocarbanilide)	Antiseptic		45%
Carbenicillin	Antibiotic	64%	46%
Oltipraz	Antitumor		46%
Bitoscanate	Anthelmintic		46%
Sarafloxacin HCl	Antibiotic		47%
Bacitracin zinc salt	Antibiotic	60%	47%
Dextrorphan tartrate	Analgesic	43%	47%
Tetramisole	Anthelmintic		48%
Bifonazole	Antifungal	50%	48%
Ethacridine lactate	Antiseptic		48%
Zanamivir	Antiviral		49%
Aluminum lactate	Antiseptic		49%
p-Arsanilic acid	Antibacterial		49%
Artemesinin	Antimalarial		49%
Nifursol	Antiprotozoal		51%

Table S1. *Cont.*

Drugs (50 µM)	Category	Residual Viable Cells (Microscopy) [b]	Residual Viable Cells (SYBR/PI) [c]
Nevirapine	Antiviral		51%
Rifaximin	Antibiotic		51%
Oxibendazole	Anthelminthic		51%
Metrifonate	Anthelmintic		51%
Indatraline hydrochloride	Monoamine transporter inhibitor	43%	51%
Florfenicol	Antibiotic		53%
Benznidazole	Antiprotozoal		53%
Ganciclovir	Antiviral		53%
Tazobactam	Antibiotic	56%	54%
Oxfendazole	Anthelminthic		54%
Phenothiazine	Anthelminthic	53%	54%
Flubendazole	Anthelminthic		54%
Midecamycin	Antibiotic		54%
Fluconazole	Antifungal	45%	55%
Docosanol	Antiviral (topical)		55%
Aztreonam	Antibiotic	50%	55%
Benzoylpas calcium (4-Benzamido salicylic acid, calcium salt)	Antibiotic		55%
Trifluridine	Antiviral (ophthalmic)		55%
Undecylenic acid	Antifungal (topical)	74%	55%
Closantel	Anthelmintic.		56%
Cefixime	Antibiotic	56%	56%
Thiamphenicol	Antibiotic		57%
Ricobendazole (Albendazole oxide)	Anthelmintic		57%
Sulfamoxole	Antibiotic	55%	57%
Clopidol	Antibacterial Coccidiostat		57%
Tosufloxacin	Antibiotic		57%
Metampicillin	Antibiotic		57%
Amikacin	Antibiotic		57%
Lamivudine	Antiviral		58%
Cephalosporin C	Antibiotic		58%
Sulfachlorpyridazine	Antibiotic		58%
Lomofungin	Antibiotic		58%
Artesunate	Antimalarial.		58%
Valacyclovir	Antiviral		58%
Carzenide (4-Carboxybenzenesulfonamide)	Antibiotic		59%
Clinafloxacin	Antibiotic		60%
Efavirenz	Antiviral		60%
Cefsulodin	Antibiotic		60%
Cloxyquin (5-chloro-8-hydroxy-quinoline)	Antibacterial		60%

Table S1. *Cont.*

Drugs (50 µM)	Category	Residual Viable Cells (Microscopy) [b]	Residual Viable Cells (SYBR/PI) [c]
Symclosene (Trichloroisocyanuric acid)	Antibacterial (topical)		60%
Didanosine (2′-3′-dideoxyinosine)	Antiviral		61%
Floxuridine (5-fluorodeoxyuridine)	Antiviral		61%
Cyacetacide	Antibacterial		61%
Roxithromycin	Antibiotic	65%	62%
Oxiconazole nitrate	Antifungal		62%
Climbazole	Antifungal	71%	62%
Protionamide	Antibacterial (tuberculostatic)		63%
Ribavirin	Antiviral		63%
Griseofulvin	Antifungal		63%
Rifamycin SV	Antibiotic	60%	63%
Salicylanilide	Antifungal (topical)		63%
Diclazuril	Antibacterial		63%
Imiquimod	Antiviral		64%
Penciclovir	Antiviral	60%	64%
Nystatin	Antifungal		64%
Ampicillin	Antibiotic		64%
Puromycin	Antibiotic	48%	65%
Stavudine (2′,3′-Didehydro-3′-Deoxythymidine)	Antiviral		65%
Potassium iodide	Antifungal		65%
Voriconazole	Antifungal		65%
Penimepicycline	Antibiotic	60%	65%
Amantadine hydrochloride	Antiviral		65%
Nitroxoline (8-hydroxy 5-nitroquinoline)	Antibiotic		66%
4-Aminosalicylic acid	Antibacterial		66%
Ciclopirox olamine	Antifungal		66%
Nelfinavir mesylate	Antiviral		66%
Anisomycin	Antibiotic		68%
Betamipron (n-benzoyl-b-alanine)	Antibacterial		68%
Famciclovir	Antiviral		68%
Flucytosine (5-Fluorocytosine)	Antifungal	66%	68%
Clotrimazole	Antifungal		68%
Rimantadine	Antiviral		68%
Pazufloxacin	Antibiotic		69%
Carbadox	Antibacterial		69%
Amantadine hydrochloride	Antiviral		69%
Dibekacin	Antibiotic		70%
Clorsulon	Anthelmintic (Trematodes)		71%

Table S1. *Cont.*

Drugs (50 µM)	Category	Residual Viable Cells (Microscopy) [b]	Residual Viable Cells (SYBR/PI) [c]
Thiacetazone (Amithiozone)	Antibacterial (tuberculostatic)		73%
Fleroxacin	Antibiotic		73%
Clofoctol	Antibiotic		73%
Butoconazole nitrate	Antifungal (topical)		74%
Quinaldine blue	Antimalarial	35%	Over range [d]
Methylene blue hydrate	Antimethemoglobinemic	40%	Over range [d]

[a] Stationary phase *B. burgdorferi* (7-day old) cells were treated with drugs for 7 days; [b] Residual viable *B. burgdorferi* was assayed by epifluorescence microscope counting; [c] Residual viable *B. burgdorferi* was calculated according to the regression equation and ratio of Green/Red fluorescence obtained by SYBR Green I/PI assay; [d]The value is higher than the drug free control.
